# Jia-Jian-Di-Huang-Yin-Zi decoction exerts neuroprotective effects on dopaminergic neurons and their microenvironment

**DOI:** 10.1038/s41598-018-27852-w

**Published:** 2018-06-29

**Authors:** Jingsi Zhang, Zhennian Zhang, Wen Zhang, Xiangting Li, Ting Wu, Tingting Li, Min Cai, Zhonghai Yu, Jun Xiang, Dingfang Cai

**Affiliations:** 1 Department of Integrative Medicine, Zhongshan Hospital, Fudan University, Shanghai, 200032 China; 2 Department of Neurology, Nanjing Hospital of Traditional Chinese Medicine, Nanjing, 210000 China; 3 Department of Neurology, Shuguang Hospital, Shanghai University of Traditional Chinese Medicine, Shanghai, 201203 China

## Abstract

As a classical prescription of Traditional Chinese medicine, the Jia-Jian-Di-Huang-Yin-Zi (JJDHYZ) decoction has long been used to treat movement disorders. The present study evaluated the effects of JJDHYZ on dopaminergic (DA) neurons and their survival-enhancing microenvironment as well as the possible mechanisms involved using a mouse model of Parkinson’s disease. In MPTP-lesioned mice, a high dosage of JJDHYZ (34 g/kg/day) attenuated the loss of DA neurons, reversed the dopamine depletion, and improved the expression of glial-derived neurotrophic factor (GDNF) compared to the untreated model group. JJDHYZ also protected the ultrastructure of the blood-brain barrier (BBB) and tight junction proteins by inhibiting the activation of microglia and astrocytes besides the increase in three types of matrix metalloproteinases in the substantia nigra. In conclusion, the JJDHYZ-high dosage (JJDHYZ-H) group exhibited the neuroprotection of DA neurons, and the underlying mechanism may be related to the survival-enhancing microenvironment of the DA neurons.

## Introduction

Parkinson’s disease is a progressive, age-related movement disorder, which is also widely known as the frequent neurological disorder of the basal ganglia. Progressive degeneration of dopaminergic neurons in the substantia nigra pars compacta (SNpc) further leads to the loss of dopaminergic neurotranmission in the striatum; following by a serious of profound functional changes of basal ganglia as well as voluntary movements^1^. Tremor, rigidity, akinesia and bradykinesia are considered as the main characteristics of movement disorders. Oxidative stress, apoptosis, neuroinflammation and mitochondrial dysfunction are the primary causes^2^. Recently, investigators have focused on the neurovascular unit (NVU), which is primarily composed of neurons and non-neuronal cells, including endothelial cells, pericytes, astrocytes, microglia and vascular smooth muscle cells. Glial cells physically play a neuroprotective role. Astrocytes envelop greater than 99% of the BBB endothelium, modulate the structure of endothelial cells and participate in the development of the BBB. In pathological condition, adverse stimulation causes glia to produce numerous neurotoxins and thereby contribute to the damage of neuroinflammation^3,4^. Activated microglia have been observed in postmortem SNpc tissues and PD models^5^. In a rat brain microvascular endothelial cells/microglia co-culture system, lipopolysaccharide (LPS) induces the activation of microglia, decreases the transendothelial electrical resistance (TEER), increases the paracellular transport of sodium fluorescein and damages the function of the BBB by producing reactive oxygen species^6,7^. TNF-α secreted by activated microglia increases the permeability of the BBB^8^, while proinflammatory cytokines, such as IL-1β, IL-4 and IFNγ, promote ICAM-1 expression to break down the normal structure of the NVU^9^. A cross-sectional analysis, comparing 38 normal elderly individuals with 100 PD patients revealed abnormal angiogenesis. Increased endothelial cells and blood vessels have been found in the SNpc area of PD patients and Parkinsonian primates^10^. BBB breakdown has also been detected^11^. Each component of the NVU seems to be closely connected and promote the progression of Parkinson’s disease. How to put off the procedure of neurodegeneration by regulating NVU is a thought-provoking question.

Pharmocological therapy has long been considered as the most efficient method for the treatment of PD. Existing treatments, like Madopar alleviate the motor symptoms but invalid in preventing neurodegeneration; dopamine agonist and MAO-B inhibitors may rise the side effects of abnormal motor output, so the study on supplemental herbs is quite essential^12^. The literature that shows the identification of patients with PD was first reported in 1,000 BC in India. The ancient civilizations used *Mucuna pruriens* to alleviate the symptoms. Recent years, natural extracts and monomers such as tannins, polyphenols, flavonoids, anthocyanins and terpenes have been extensively investigated in the medical sciences^13^.

Traditional Chinese medicine (TCM) has been used clinically for more than 3000 years. The Jia-Jian-Di-Huang-Yin-Zi decoction (JJDHYZ), which derives from a classical prescription in treating motor and language disorder, was effective in our previous clinical trials wherein both the Unified Parkinson’s Disease Rating Scale (UPDRS) scores and the Madopar dosage were reduced in PD patients^14^. *Radix Rehmanniae*, *Fructus Corni*, *Radix Morindae Officinalis*, *Herba Cistanches*, *Radix Angelicae Sinensis*, *Radix Asparagi*, *Radix Paeoniae Alba* are included in the prescription with a ratio of 1:0.6:1:1:1:1:1. And the main chemical compounds of these crude herbs are: Rubiadin (PubChem CID: 124062); Paeoniflorin (PubChem CID: 442534); Albiflorin (Pubchem CID: 24868421); Ferulic acid (Pubchem CID: 445858); Catalpol (Pubchem CID: 91520); Verbascose (Pubchem CID: 441434); Cistanoside D (PubChem CID: 5315930); Echinacoside (PubChem CID: 5281771); Rehmannioside A (PubChem CID: 6325881); Rehmannioside D (PubChem CID: 92044472) and Ursolic acid (PubChem CID: 64945). JJDHYZ showed behavior recovery and dopaminergic neurons protection by inhibiting apoptosis associated with mitochondrial and ER caspase 12 pathways in our previous study^15^. In the present study, we mimicked PD symptoms in a subacute mouse model using MPTP and investigated whether JJDHYZ played a regulatory role regarding DA neurons and their surrounding milieu.

## Results

### JJDHYZ-high dosage (JJDHYZ-H) improved the expression of glial-derived neurotrophic factor (GDNF) in MPTP-lesioned mice

**Figure 1:**
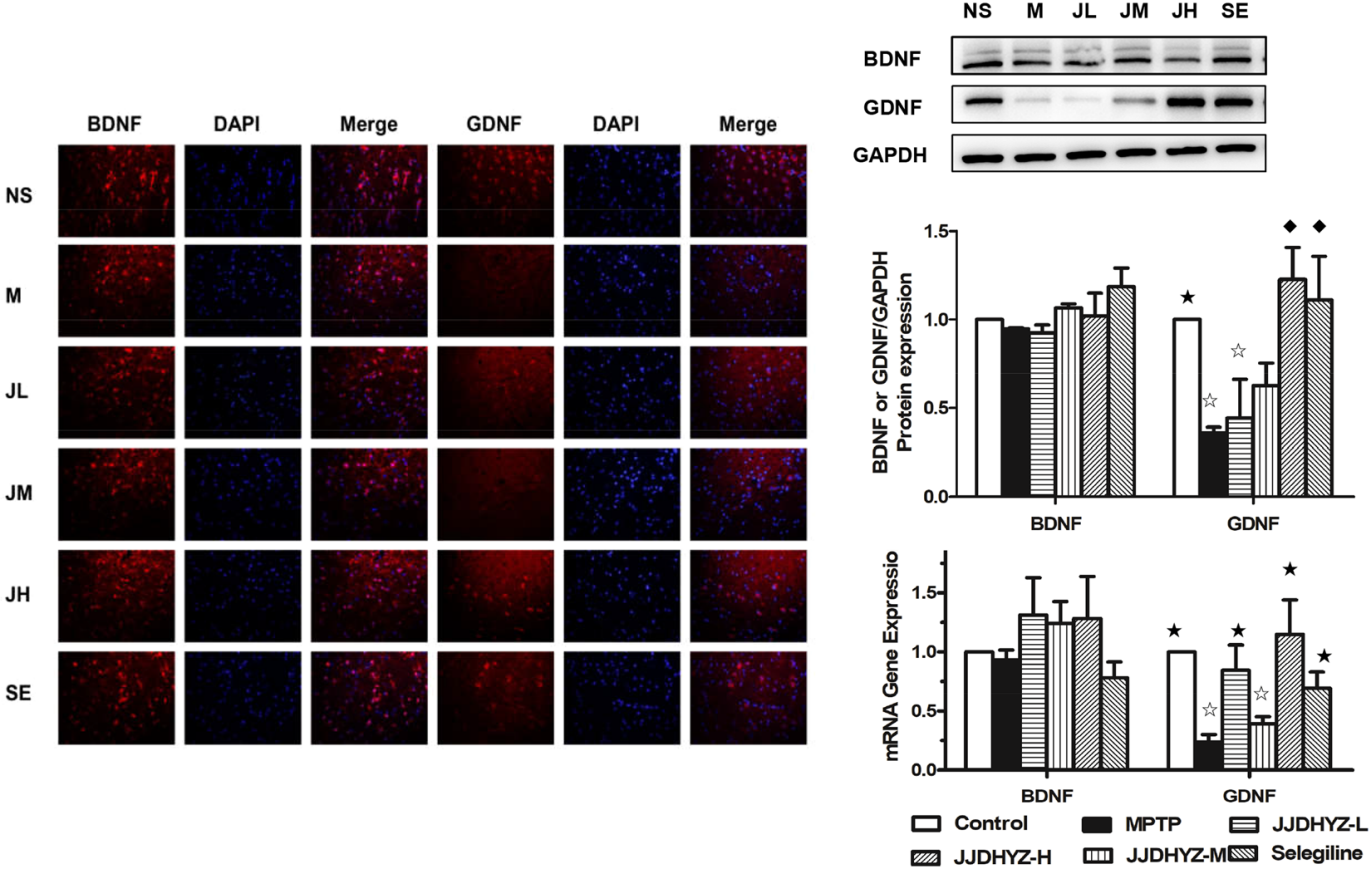
Immunofluorescence, real-time PCR and Western blot analysis of BDNF and GDNF in the SNpc. (**A**) BDNF- and GDNF-immunopositive cells in the different groups: NS (control), M (MPTP), JL (JJDHYZ-L), JM (JJDHYZ-M), JH (JJDHYZ-H) and SE (selegiline). Both BDNF and GDNF are stained red, nuclei are stained blue; the positive cells are indicated by red merged with blue. (**B**,**C**) Protein and Gene expressions of BDNF and GDNF in each group. Full-length blots are presented in Supplementary Figure 1’. Images were captured at ×400 magnification. Positive cells and protein expressions were analyzed by Image J software. Three animals were used for each experimental group. ^☆^*P* < 0.05, ^◇^*P* < 0.01 vs. the control group; ^★^*P* < 0.05, ^⬩^*P* < 0.01 vs. the MPTP group.

In our previous study, JJDHYZ-H (34 g/kg/day) has been verified as the optimal dosage to ameliorate the behavior performance of PD mice, reduce the loss of DA neurons and restore the content of dopamine and its relevant metabolites^15^. To further clarify the neuroprotective role of JJDHYZ, we assessed the expressions of brain-derived neurotrophic factor (BDNF) and GDNF using immunofluorescence, real-time PCR and Western blot analysis. No significant differences were observed in the numbers of BDNF-positive cells, BDNF gene or protein expression among the groups (Fig. 1). However, MPTP significantly decreased the numbers of GDNF-positive cells (*P* < 0.01, Fig. 1A), GDNF mRNA and protein expression compared to that of the control group (*P* < 0.05, Fig. 1B,C). JJDHYZ-H and selegiline reversed the loss of GDNF-positive cells and expression compared to those of the MPTP-lesioned group (*P* < 0.01). Although JJDHYZ-L improved the levels of gene expression of GDNF (*P* < 0.05, Fig. 1C), no neuroprotective effect was detected using immunostaning and western blot (Fig. 1A,B).

### JJDHYZ protected antioxidant enzyme activity

**Figure 2:**
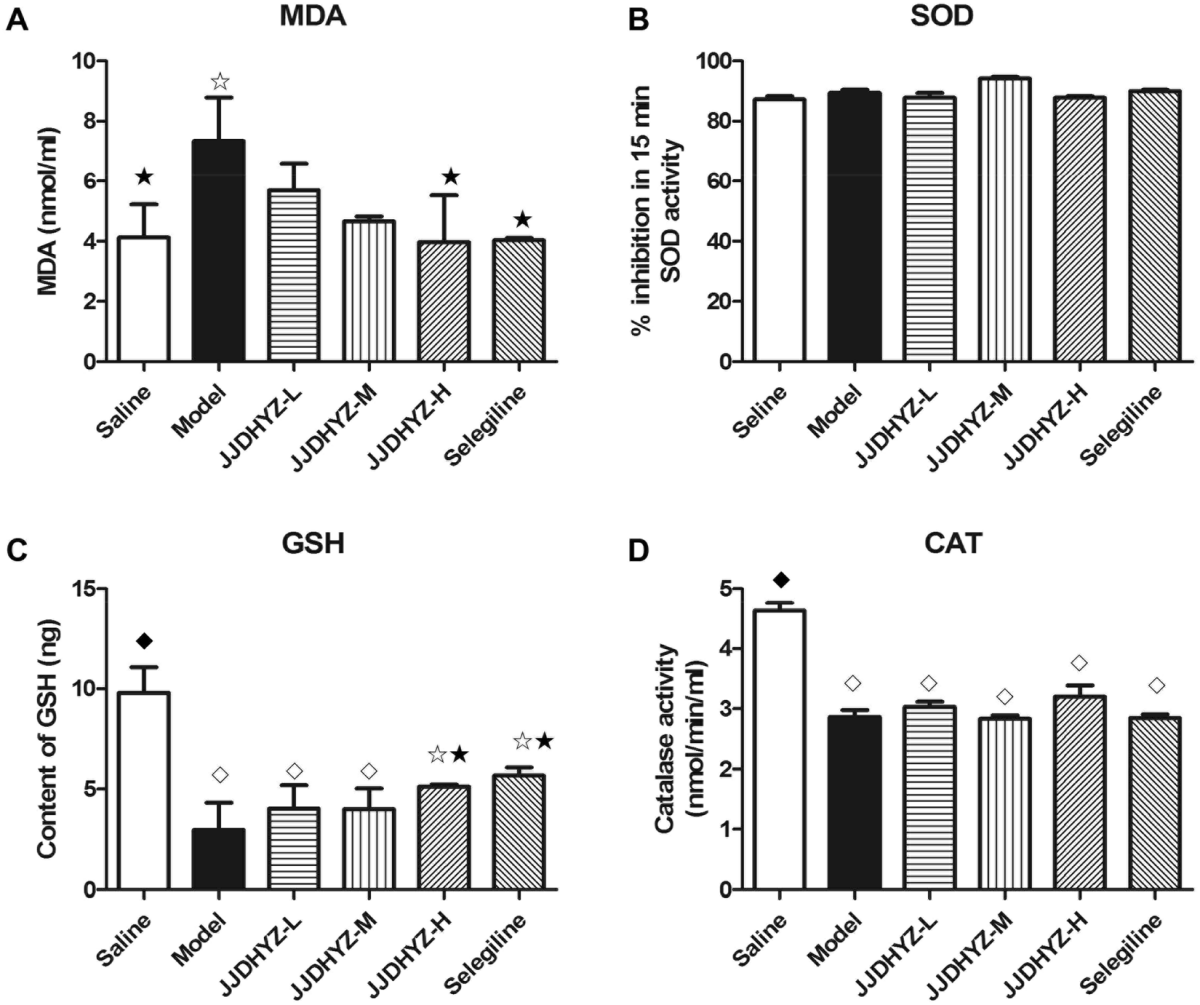
Estimation of MDA, SOD, GSH and CAT in the SNpc. The MPTP injection induced an increase in MDA (**A**) with a decrease in both GSH (**C**) and CAT (**D**) in the SN compared with those in control group. Selegiline and a high dosage of JJDHYZ, inhibited the production of MDA and increased the level of GSH to a certain extent. Changes in SOD (**B**) were not observed. 3 animals were used for each experimental group. ^☆^*P* < 0.05, ^◇^*P* < 0.01 vs. the control group; ^★^*P* < 0.05, ^⬩^*P* < 0.01vs. the MPTP group.

The activity of the antioxidant enzymes superoxide dismutase (SOD), catalase (CAT), and glutathione (GSH) was measured. We also determined the concentration of malondialdehyde (MDA) to assess membrane lipid peroxidation. GSH (Fig. 2C) and CAT (Fig. 2D) were decreased significantly (*P* < 0.01) in the MPTP-lesioned mice, compared to the control animals. JJDHYZ-H and selegiline increased GSH activity and reduced MDA production compared to the model group (*P* < 0.05, Fig. 2A,C). No significant difference was observed in SOD (Fig. 2B). Neither JJDHYZ nor selegiline affected the rescuing activity of CAT after MPTP injection.

### JJDHYZ-H inhibited the activation of microglia and astrocytes in the SN

**Figure 3:**
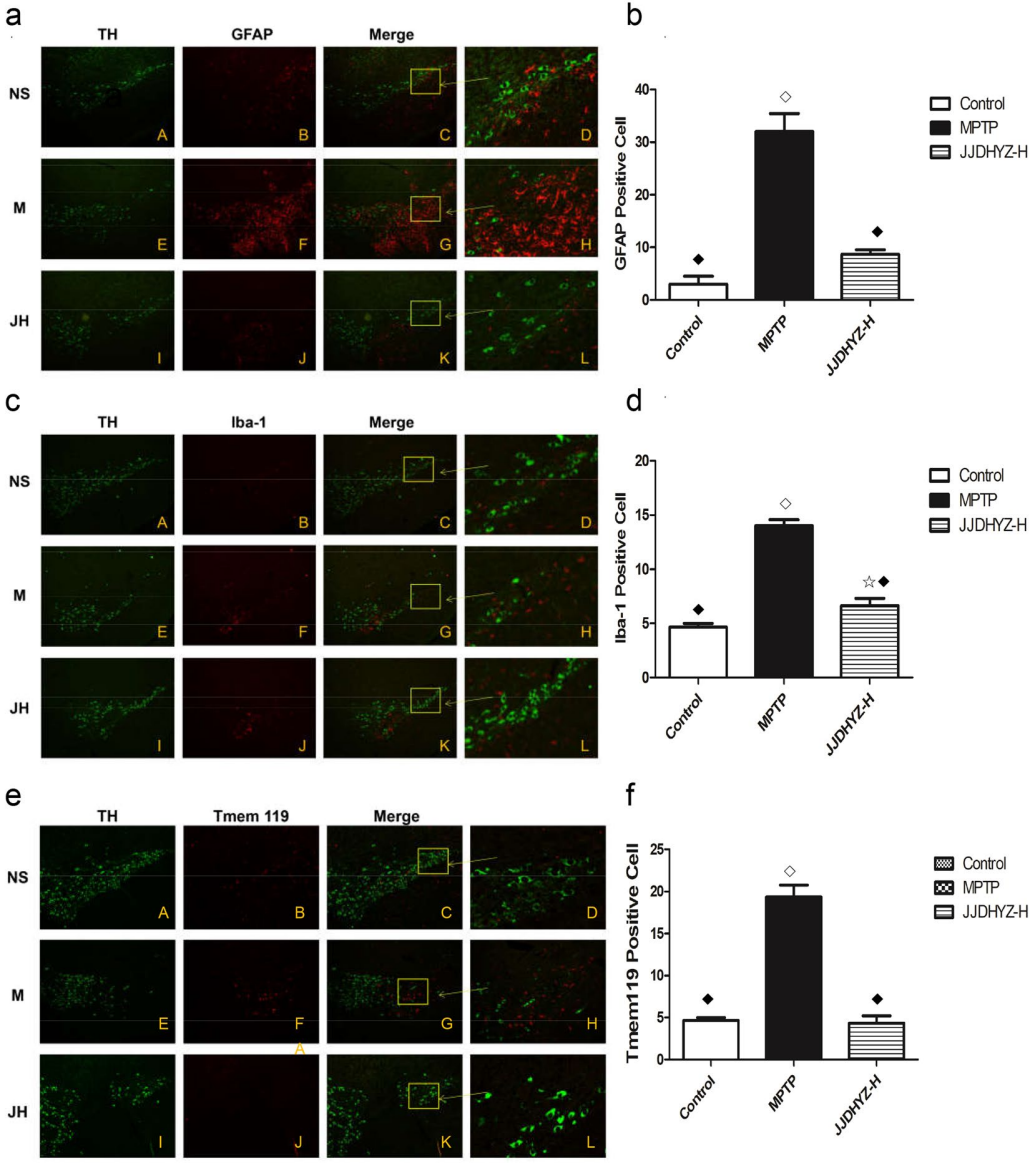
Double immunostaining of dopaminergic neurons with microglia or astrocyte. GFAP was used as the biomarker of astrocyte, while Iba-1 and Tmem119 were used to mark the microglia. Panels a, c and e depict double immunostaining of TH with activated GFAP, Iba-1 or Tmem119 respectively. Activated glial cells are labeled with Cy3 (red), and the DA neurons are stained green in the control (NS), model (M or MPTP) and JJDHYZ-H (JH) groups. Images were first observed with ×100 magnification (A–K); D, H and L are enlargements of the rectangular areas marked in C, G and K. Few activated astrocytes and microglia were observed in the control and JJDHYZ-H groups, while MPTP promoted an increase in the number of these cells (*P* <0.001). Histograms showing the number of GFAP-positive cells are presented in panel b. Bar graphs of Iba-1-positive cells and Tmem119-positve cells are presented in panel d and f. ^☆^*P* <0.05, ^◇^*P* <0.01 vs. the control group; ^★^*P* <0.05, ^⬩^*P* <0.01 vs. the MPTP group.

We detected microglial and astrocyte activation in the substantia nigra of the control, model and JJDHYZ-H groups. GFAP was used as the biomarker of astrocyte, while Iba-1 and Tmem119 were used to mark the microglia. Few glial cells, indicators of neuroinflammation, were observed under normal, physical condition (A–C in Fig. 3a,c,e). However, numerous microglia and astrocytes were activated after MPTP administration in the model group (E-G). JJDHYZ-H invention inhibited the activation of microglia and astrocytes to some extent compared to those in the model group, although the results were still different from the control group (I-J).

### JJDHYZ-H attenuated the ultrastructural changes in the BBB

**Figure 4:**
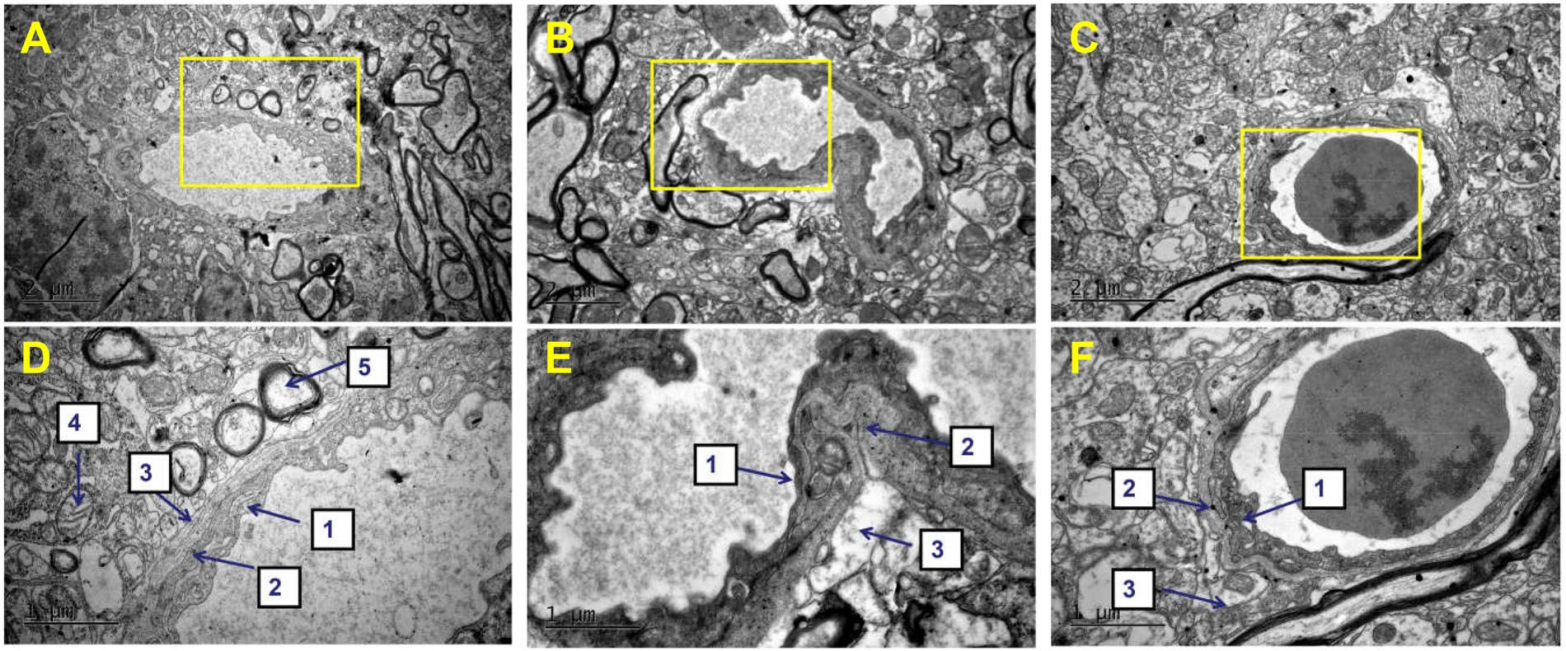
Ultrastructure of the BBB in the midbrain. (**A**–**C**) Ultrastructure of the BBB in the control, MPTP, and JJDHYZ-H groups, respectively. (**D**) Enlargement of the yellow box in (**A**) showing the integrity of the basal membrane, normal tight junctions, endothelial cells, and astrocytic end-feet. (**E**) Enlargement of the boxed region in (**B**); MPTP induced the swelling of astrocytic end-feet, the disruption of the endothelium and deflated microvessels. (**F**) Enlargement of the boxed region in C (JJDHYZ-H), with a clear ultrastructure of the BBB similar to that in the control group. The number 1stands for endothelial cells, 2 extracellular matrix, 3 astrocytic end-foot, 4 mitochondrial, 5 lysosome. 3 Animals were used for each experimental group.

The neuroprotective effects of JJDHYZ-H may potentially correlate with the changes in the BBB in subacute mouse models of PD. BBB ultrastructural alterations were detected using a transmission electron microscope (TEM). Microvessels in the SN of the control group were normal with a continuous basal lamina, astrocytic end-feet and normal endothelial cells (Fig. 4A). However, swollen endothelial cells and astrocytic end-feet, deflated capillary lumens and edema surrounding the microvessels were distinctly visible in the MPTP-lesioned group (Fig. 4B). JJDHYZ-H alleviated the swelling of the astrocytic end-feet. Microvessels with a continuous basal membrane and integrity of the endothelium were also observed (Fig. 4C).

### JJDHYZ-H maintained claudin-5 and occludin expression and BBB permeability in the SNpc

**Figure 5:**
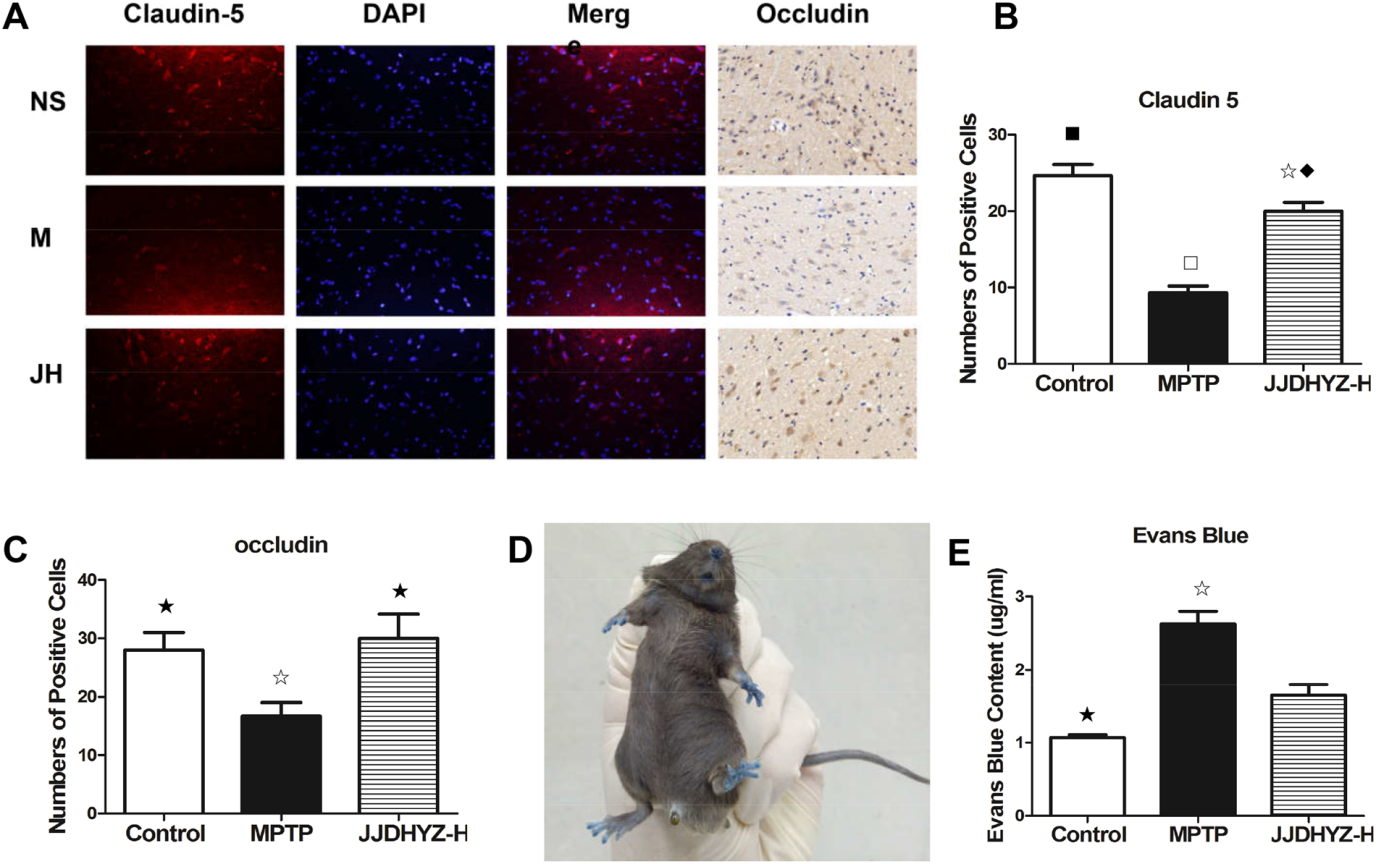
Expressions of claudin-5- and occludin-positive cells and BBB permeability in the SNpc. (**A**) Claudin-5 and Occlucin expressions in the different groups: NS (control), M (MPTP), and JH (JJDHYZ-H) groups. Claudin-5 is stained red, and nuclei are stained blue; the positive cells are indicated by red merged with blue. The nuclei of occludin are stained blue, and positive cells are indicated by a brown color. (**B**), (**C**) Histograms of claudin-5- and occludin-positive cell numbers. (**D**) A c57BL mouse after Evans Blue injection. (**E**) The concentration of Evans Blue in the SNpc tissues from each group. Images were captured at × 400 magnification. Five animals were used for each experimental group. ^☆^*P* < 0.05, ^◇^*P* < 0.01, ^□^*P* < 0.001 vs. the control group; ^★^*P* < 0.05, ^⬩^*P* < 0.01, ^■^*P* < 0.001 vs. the MPTP group.

We investigated the effect of JJDHYZ-H on two significant transmembrane tight junction proteins, claudin-5 and occludin. An immunostaining analysis of claudin-5 and occludin revealed changes in expression following the MPTP injection. A significant loss of claudin-5- and occludin-positive cells was found in the SNpc (*P* < 0.001, *P* < 0.05, respectively, Fig. 5B,C), and this loss was associated with an increase in BBB permeability (*P* < 0.05, Fig. 5E) in the MPTP group compared to that in the control group. JJDHYZ-H ameliorated the loss of both claudin-5 (*P* < 0.01) and occludin (*P* < 0.05), but no obvious changes in BBB permeability were observed using the Evans Blue method.

### JJDHYZ-H reduced the abnormal increase in microvessels in the SNpc

**Figure 6:**
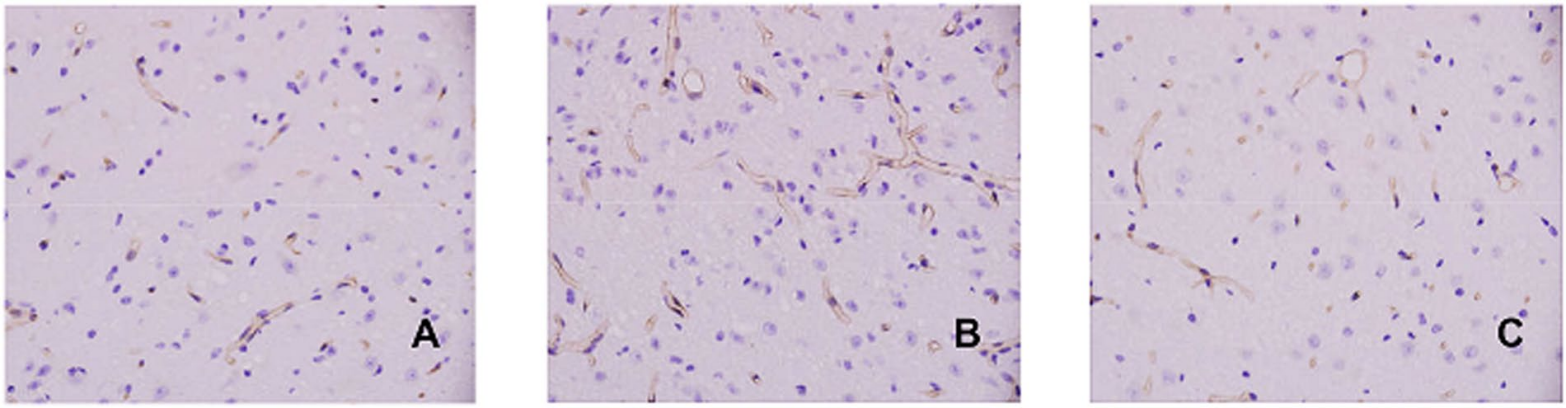
CD31(+) microvessel immunostaining in the SN. (**A**–**C**) Expressions of CD31(+) microvessels in the control, model and JJDHYZ-H groups, respectively. Images were captured at ×200 magnification.

CD31 was used to identify microvessels. We found that there were few CD31(+) microvessels in the SN of the control group (Fig. 6A). However, MPTP administration significantly increased the occurrence CD31(+) microvessels (Fig. 6B), and JJDHYZ-H reduced the number of abnormal microvessels compared to that in the model group (*P* < 0.05).

### JJDHYZ-H down-regulated matrix metalloproteinase (MMP)2, MMP3 and MMP9 expression in the substantia nigra

**Figure 7:**
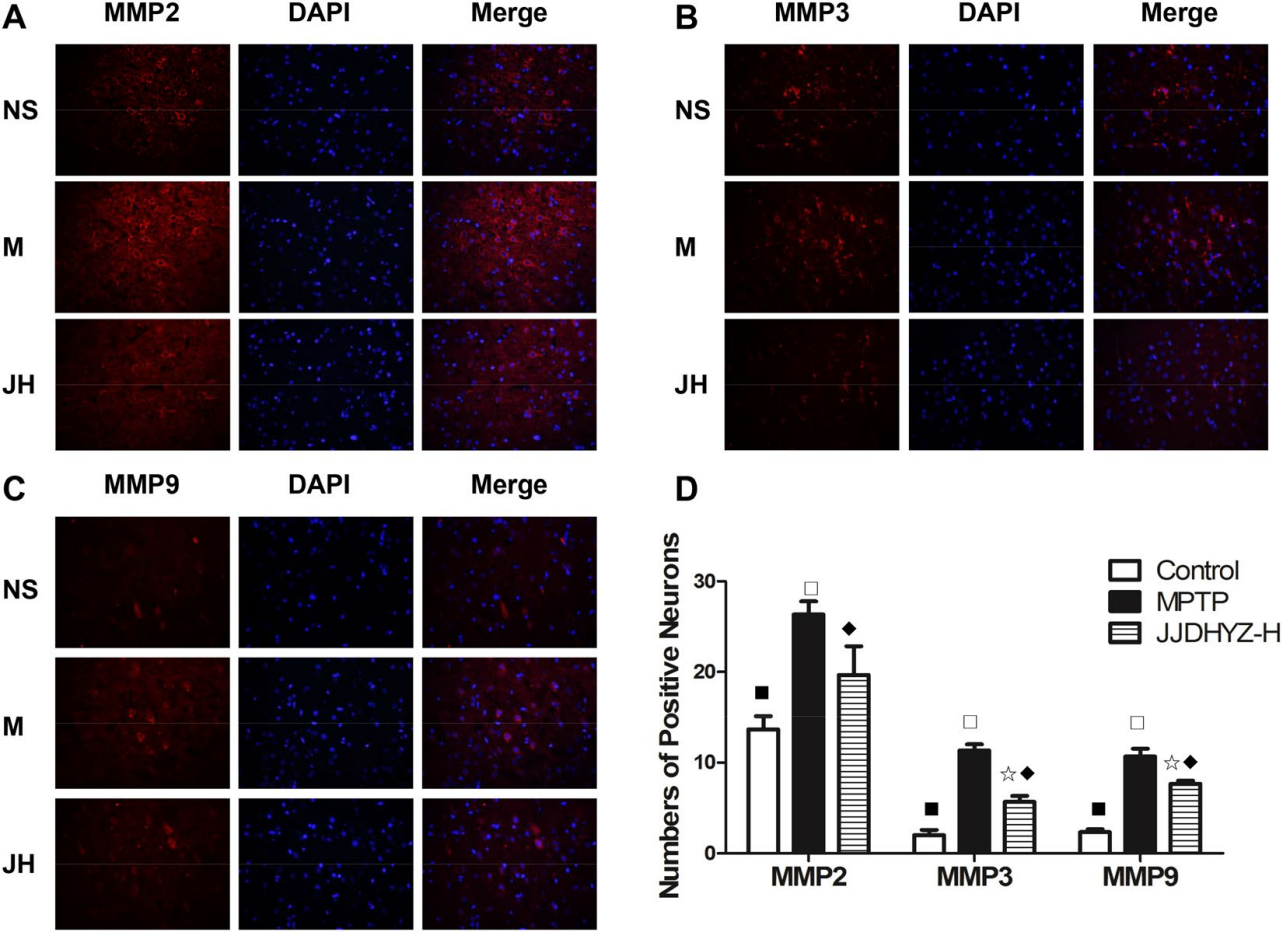
MMP2, MMP3 and MMP9 expression in the substantia nigra. (**A**) MMP2-positive cells in the substantia nigra of the control (NS), model (M) and JJDHYZ-H (JH) groups. (**B**) MMP3(+) expression in each group. (**C**) MMP9(+) expression in each group. MMPs were labeled using Cy3 (red), nuclei are stained with 4′,6-diamidino-2-phenylindole (DAPI) (blue); positive cells are indicated by red merged with blue. (**D**) Histograms of MMP expression. Images were captured at ×400 magnification. 5 Animals were used for each experimental group. ^☆^*P* < 0.05, ^◇^*P* < 0.01, ^□^*P* < 0.001 compared with the control group; ^★^*P* < 0.05, ^⬩^*P* < 0.01, ^■^*P* < 0.001 compared with the MPTP group.

MMP2, MMP3 and MMP9 were labeled via immunofluorescence (Fig. 7). The expression of all three of these MMPs was increased in the model group compared with that in the control group (*P* < 0.001) and the optimal JJDHYZ group (*P* < 0.01). Although down-regulation was observed, MMP3 and MMP9 expression was nevertheless different between the JJDHYZ-H and the control group (*P* < 0.05, Fig. 7D).

### JJDHYZ-H regulated the expressions of IL-23, CCL2 and CCL4

**Figure 8:**
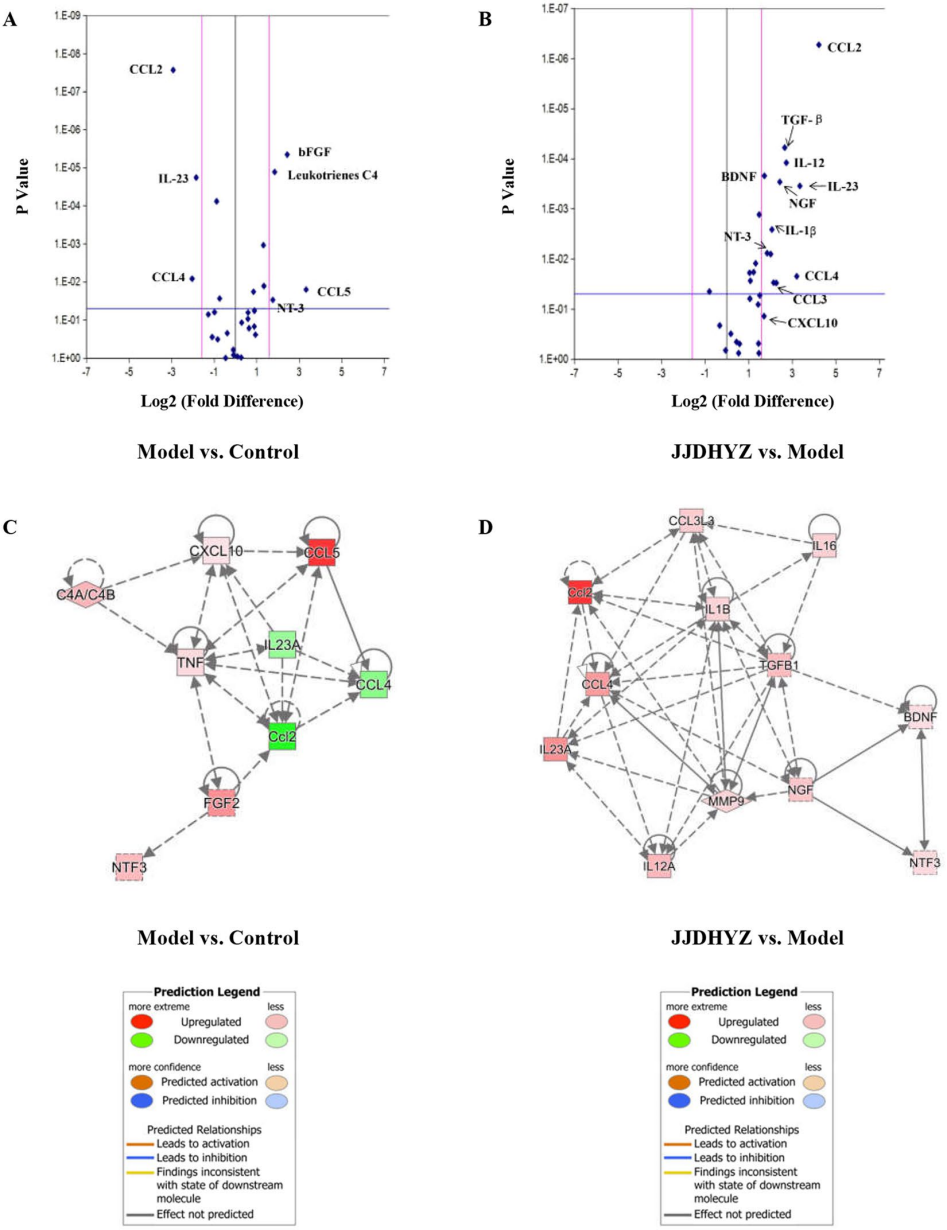
Volcano Plot and Ingenuity Pathway Analysis (IPA) of 29 genes. Panel (A) and (B) are volcanno plot. Each blue point represents a gene. The x-coordinate stands for the fold-change of the gene expression and the y-coordinate is the *p*-value from the statistic analysis. Panel (A): Changes of gene expressions between the model group and control group, expressions of bFGF, Leukotrinens C4, CCL5 and NT-3 were up-regulated in MPTP-lesioned mice (*P* < 0.05), while CCL2, CCL4 and IL-23 were down-regulated (*P* < 0.05). Panel (B): JJDHYZ-H up-regulated the expressions of CCL2, TGF-β, BDNF, IL-12, IL-23, NGF, IL-1β, NT-3, CCL3, CCL4 and CXCL10. The genetic interaction network (Panel C & D) showed the molecular interactions. Similar to the volcano plot, only those genes with fold-change (FC) >2 or <−2 and *p*-value < 0.05 were included. Red (gene up-regulated), green (down-regulated). The more significant changed genes were marked with the darker colors. The lines and arrows annotated the types of molecular interactions. Interactions which have not been predicted are marked with the gray color.

Volcano plots are commonly used to analyze the mRNA expressions of microarray. Each point in the scatter plot represents a gene^16^. Based on published manuscripts and reviews, we listed the mostly studied 29 factors in Table 1, analyzed their gene expressions with PCR array and presented the result with volcano plots. The genes with fold-change (FC) >2 or <−2, besides *p*-value < 0.05 were considered as significant changed, and marked as the blue points out of the two perpendicular lines (Fig. 8A,B). Compared with the control group, MPTP model group down-regulated the expressions of CCL2, CCL4 and IL-23 as well as improved the expressions of CCL5, bFGF, NT-3 and Leukotrinens C4 (Fig. 8A). Similar to the control group, JJDHYZ-H reversed the inhibitory effects of MPTP on CCL2, CCL4 and IL-23; but it also up-regulated the other 8 cytokines’ expressions (Fig. 8B). Then the gene changes were processed with ingenuity pathway analysis (IPA). IPA maps molecules from the Ingenuity Pathway Knowledge Base. The computing methods of fold-changes of per gene were similar to the volcano plot. Statistical significant difference was assessed by Right-Tailed Fisher’s Exact Test^17^. The genetic interaction network (Fig. 8C,D) showed the molecular interactions under this experimental condition. In general, red color stands for the up-regulation of genes, green stands for the down-regulation. The more significant changed genes were marked with the darker colors. The lines and arrows annotated the types of molecular interactions. Interactions which have not been predicted are marked with the gray color. We verified the result with a multi-analyte ELISA array. Expressions of CCL2, CCL4 and IL-23 were significantly inhibited in the model group (*P* < 0.05), while no statistical difference were observed among the other cytokines or chemokines.

**Table 1:** PD relevant cytokines and chemokines.

	Pro-neuroinflammation	Anti-neuroinflammation
Cytokines	IL-1β, IL-2, IL-6, IL-8, IL-12, IL-16, TNF-α, INF-α	IL-4, IL-10, IL-23, PGE2
Chemokines	CCL2, CCL3, CCL4, CCL5, CXCL9, CXCL10, CXCL12, CX3CL1	
Others	PGD2, Leukotrinens C4	BDNF, GDNF, NGF, NT-3, NT-4, TGF-β, bFGF

Note: CCL2 (MCP-1), CCL3 (MIP-1α), CCL4 (MIP-1β), CCL5 (RANTES), CXCL9 (MIG), CXCL10 (IP-10), CXCL12 (SDF-1), CX3CL1 (fractalkine).

## Discussion

Current treatments of PD mainly aim to alleviate clinical symptoms. As one of the potential intervening methods, neurotrophic factors play the roles of nourishing neurons, prompting axon regeneration^18^. The decrease of neurotrophic factors may induce the degeneration of dopaminergic neurons. Scientists immunostained the brain tissues of PD and non-PD patients aging 72–79 years to observe the expressions of various neurotrophic factors. Almost all the factors were decreased in some extend and the loss of GDNF was most outstanding^19^. Additionally, GDNF alleviated patients’ motor symptoms by putamen injection^20^, as well as promoted the survival of dopaminergic neurons *in vitro*^21^. In present study, we chose BDNF and GDNF to evaluate the neurotrophic effects of JJDHYZ decoction and observed that JJDHYZ in high dosage involved in up-regulating GDNF. Actually, the neuroprotective effects of JJDHYZ on PD mice were more than that.

Central nervous system is particularly sensitive to oxidative stress for its high oxygen consumption, high production of reactive oxygen and increasing deposition of neurotoxic metal irons^22^. Physically, antioxidant enzymes such as suoeroxide dismutase (SOD), glutathione peroxidase (GPx), and catalase (CAT) catalyze free radicals. However, the auto-oxidation and enzymatic metabolism of dopamine leads to the production of neurotoxic free radicals and excessive oxygen species. Accumulated free radicals trigger oxidative stress to damage the biological molecules in cells and cause cell death^23^. Postmortem studies have revealed the reduction of GSH and increment of GSSG in the SNpc, which weaken the natural antioxidant defense and trigger the degeneration of nigral neurons^24^. In animal models, neurotoxin as MPTP induces the generation of free radicals and aggravated the burdens of antioxidant enzymes^25^. In our present study, we observed that MPTP inhibited the expressions of GSH, CAT and improved the concentration of MDA. JJDHYZ inhibited the generation of MDA, rescued the loss of the antioxidant GSH, especially at the high dosage.

With the in-depth study on PD, neuroinflammation is also considered to be associated with the selective loss of dopaminergic neurons. Therefore, the therapeutic intervene on microglia and astrocytes activation would be one of the effective strategies. Microglia are important immunity monitors that form the first defensive line against invading pathogens and rapidly respond to CNS injuries. Excessive microglia and astrocytes have been observed in the substantia nigra and striatum of autopsy cases^26^. Activated microglia and astrocytes generate ROS^27^ and release proinflammatory cytokines, chemokines and other proteinases^28^. The levels of IL-1β, IL-2, IL-6 and TNF-α are increased in the cerebrospinal fluid of patients with familial or sporadic PD^29^. Similar observations have been reported in MPTP- and 6-OHDA- induced animal models. These secreted factors promote alternations in the neuronal milieu and increase the neuronal susceptibility to death^30,31^. We double immunostained the GFAP with TH to detect activated astrocytes in the SN. In the meanwhile, we used two biomarkers to label microglia. Besides the well-known biomarker Iba-1, the other new cell-surface, microglia-specific marker named transmembrane protein 119 (Tmem119) was used^32^. The results demonstrated a significant loss of TH-positive cells concomitant with increased numbers of glial cells in the model group (Fig. 3). JJDHYZ-H treatment inhibited the loss of DA neurons and destructive neuroinflammation.

Through the results mentioned above, we primarily draw the conclusion that JJDHYZ had neuroprotective effects on PD mice. But the mechanisms were still uncertain. As the survival of dopaminergic neurons depends on the microenvironment, the potential connections between neurons and NVU attracted us most, so we further explored the regulated effects of JJDHYZ on NVU.

NVU is primarily composed of neurons and non-neuronal cells, including endothelial cells, pericytes, astrocytes, microglia and vascular smooth muscle cells. BBB is the key component. First, we observed the ultrastructural changes in the BBB by TEM. Normal microvessels were composed with continuous basal lamina, astrocytic end-feet and normal endothelial cells, which can be clearly observed under a transmission electron microscope. However, in MPTP-induced model, normal structures were instead by swollen endothelial cells and astrocytic end-feet, deflated capillary lumens and edema (Fig. 4B). JJDHYZ-H alleviated the swelling of the astrocytic end-feet. Microvessels with a continuous basal membrane and integrity of the endothelium were also observed (Fig. 4C). Angiogenesis is an important mechanism involved in the pathophysiology of PD^33^. Increased numbers of endothelial cell nuclei and blood vessels have been reported postmortem in the SNpc of patients with PD and prakinsonian primates. The increment of CD31 may related to the disruption of blood brain barrier and neuroinflammation^11,34,35^. For instance, MPTP-induced mice with TNF-α knockout were more possible to avoid the disruption of BBB^36^. Herein, we examined the BBB, neurons and glial cells in combination to deeply explore this treatment. Microvessels were labelled with an anti-CD31 antibody and more CD31-positive microvessels were found in the model group (Fig. 6, *P* < 0.05), which is in according with the reports of angiogenesis in PD^33,35,37^.

Microvessels regulate the exchanges of substances inside and outside the vessels and form a natural barrier to protect CNS. The tight junction proteins, which tightly connected are high selectivity to restrict the entrance of exogenous substances through the cell gap. We detected occludin and claudin-5 expression in the SNpc via immunostaining (Fig. 5). MPTP depleted these two tight junction proteins, while the proteins in the JJDHYZ-H-treated mice were not significantly different from those in the normal group. In addition to tight junctions, alternations of MMPs also influence the function of the BBB^38^. These zinc-dependent enzymes are key mediators of tight junction protein alterations, and exhibit proteolytic activity on the extracellular matrix, including the basal lamina in the NVU^39^. Under neuroinflammatory conditions, MMP3 and MMP9 are first secreted in response to inflammatory injury, and MMP2 is secreted in a zymogen form, which is activated upon host injury in the CNS^40^. All three of these MMPs cleave tight junction proteins. Occludin is vulnerable to attack by MMPs, MMP-2 and MMP-9 degrade claudin-5 after ischemic insult^41^. MMPs also degrade basal lamina proteins, such as fibronectin, laminin and heparan sulfate, after an ischemic insult, which contributes to BBB breakdown^42,43^. Several reports claimed that MMPs are involved in apoptosis. Dead DA neurons release MMP3 *in vitro*, which further induces microglial activation^44^. We fluorescently stained MMP2, MMP3 and MMP9, and determined that the expressions of all three of these MMPs was increased in the model group (Fig. 7). JJDHYZ-H maintained the expression of claudin-5 and occluding, probably by inhibiting the activation of MMPs.

To study the microenviromental changes of immune factors, we selected 29 factors from published articles, each of them was closely related with microglia and astrocytes. Compared to the control group, CCL2, CCL4 and IL-23 were down-regulated in the model group. JJDHYZ at high dosage reversed the inhibitory effects of MPTP on CCL2, CCL4 and IL-23 (Fig. 8). Increased CCL2-mRNA expression has been reported in an acute PD model induced by MPTP, and CCL2 may further induce the expression of CCL4; however, SNpc neuronal loss is not significant reduced in CCL2-knockout mice^45^. The role of CCL2 in PD is still uncertain. IL-23 is produced by myeloid cells and has been considered as one of the key cytokines for targeted therapies in immune-mediated diseases^46,47^. Based on former reports, CCL2 and CCL4 were thought to promote inflammation in the models of Alzheimer’s disease, while IL23 inhibits inflammation. Does this paradoxical with the neuroprotective effects of JJDHYZ? Since the role of these cytokines are unclear in PD, underlying mechanisms need to be investigated. The reason why JJDHYZ up-regulated CCL2,CCL4 and IL23 is still exploring and we will share the results in the future.

## Materials and Methods

### Ethics statement

The present study was performed according to the Guidelines for the Care and Use of Laboratory Animals from the National Institutes of Health. The Committee for Animal Research of Fudan University approved all procedures.

### Animals

Male c57BL/6 mice weighing 22–25 g (aging 8–10 weeks) were housed in a temperature-controlled environment (constant temperature of 22 ± 3 °C) with 60% humidity and a 12-h light/dark cycle. Food and water were available *ad libitum*.

### Preparation of JJDHYZ

The JJDHYZ decoction is composed of seven crude herbs: *Radix Rehmanniae, Fructus Corni, Radix Morindae Officinalis, Herba Cistanches, Radix Angelicae Sinensis, Radix Asparagi* and *Radix Paeoniae Alba* at a ratio of 1:0.6:1:1:1:1:1 (details about the herbs could be seen in Table 2). All the herbal components were purchased from Zhongshan Hospital, Fudan University. All crude herbs were first immerged in distilled water for 30 min and boiled for 1 h, and collected the suspension. Distilled water was then added into the herbal residues and the process was repeated twice. Dehydrated alcohol was added in the collected suspension to a final concentration of 75% alcohol (v/v). The residues were soaked in 75% ethyl alcohol for 24 h to collect the suspension. The suspension and liquid acquired from the residues were mixed, and centrifuged at 2,000 g for 20 min. The alcohol was completely volatilized by a rotary evaporator. The final concentration of the JJDHYZ decoction was 1 g/ml (w/v).

**Table 2:** Herbs in Jia-Jian-Di-Huang-Yin-Zi Decoction.

Chinese name	English name	Latin name	Used part
Di Huang	Radix Rehmanniae	Rehmannia Glutinosa Libosch	Root
Shan Zhu Yu	Fructus Corni	Cornus Officinalis Sieb.et Zucc	Fruit
Ba Ji Tian	Radix Morindae Officinalis	Morinda Officinalis How	Root
Rou Cong Rong	Herba Cistanches	Cistanche Deserticola Y.C.Ma	Stem
Dang Gui	Radix Angelicae Sinensis	Angelica Sinensis (Oliv.) Diels	Root
Tian Dong	Radix Asparagi	Asparagus Cochinchinensis Merr.	Root
Bai Shao	Radix Paeoniae Alba	Paeonia Lactiflora Pall.	Root

Notes: seven herbs listed in the table is at a ratio of 1:0.6:1:1:1:1:1.

### Chemical compounds of JJDHYZ

The JJDHYZ contains rubiadin (PubChem CID: 124062), paeoniflorin (PubChem CID: 442534), albiflorin (Pubchem CID: 24868421), ferulic acid (Pubchem CID: 445858), catalpol (Pubchem CID: 91520), verbascose (Pubchem CID: 441434), cistanoside D (PubChem CID: 5315930), echinacoside (PubChem CID: 5281771), rehmannioside A (PubChem CID: 6325881), rehmannioside D (PubChem CID: 92044472), and ursolic acid (PubChem CID: 64945).

### Study design

C57BL/6 mice were orally administered saline, JJDHYZ or selegiline for 14 days followed by an intraperitoneal injection of saline or MPTP (30 mg/kg/day for 5 days). The mice were randomly divided into the following six groups: (1) control group: saline injection plus saline gastrointestinal administration; (2) MPTP group: MPTP injection plus saline; (3) JJDHYZ-low dosage group (JJDHYZ-L): MPTP injection plus JJDHYZ 8.5 g/kg/day; (4) JJDHYZ-middle dosage group (JJDHYZ-M): MPTP injection plus JJDHYZ 17 g/kg/day; (5) JJDHYZ-high dosage group (JJDHYZ-H): MPTP injection plus JJDHYZ 34 g/kg/day; and (6) selegiline group: MPTP injection plus selegiline 1.0 mg/kg/day^48^. MPTP powder and selegiline were solubilized in saline.

### Immunohistochemistry

Sections of the SNpc (−3.64 mm~−2.92 mm from Bregma) were obtained from each mouse. The sections were dewaxed, hydrated, and washed with phosphate-buffered saline (PBS) (5 min × 3), and antigen retrieval was then performed in citric acid buffer (pH 6.0). Endogenous peroxidase activity was blocked with a 3% hydrogen peroxide-methanol buffer. Following incubation with 10% goat serum at room temperature for 1 h, the sections were incubated with an anti-occludin antibody (ab168986, Abcam) at 4 °C overnight. Subsequently, the immunolabeling was continued using a horseradish peroxide (HRP) secondary antibody (goat anti-rabbit, A0208, Beyotime). Immunopositive cells were counted from 5 slides of the SN region per mouse and the average was taken. 3 animals per group were used. All positive cell numbers were counted in SNpc under 40 magnification using the Image Pro Plus 6.0 software (Media Cybernetics, USA).

### Immunofluorescence

Frozen sections of the SNpc were fixed in acetone for 20 min, treated with 3% hydrogen peroxide in methanol, and then incubated with a 10% goat serum blocking solution. Next, the sections were incubated at 4 °C overnight with antibodies against the following target proteins: BDNF **(**ab108319, Abcam), GDNF (ab18956, Abcam), Iba-1 (#019–19741, woko, Japan), Tmem119 (ab209064, Abcam), claudin-5 (ab15106, Abcam), MMP2 (ab92536, Abcam), MMP3 (ab53015, Abcam), and MMP9 (ab76003, Abcam). A Cy3-conjugated goat anti-rabbit antibody (A0516, Beyotime) was used as the secondary antibody. Nuclei were counterstained with DAPI (C1005, Beyotime). Images of the positively stained cells were captured using a fluorescence microscope (Olympus BX51, Japan). The quantification method was same as immunohistochemistry.

### Quantitative RT-PCR analysis

Total RNA from SNpc tissues was extracted using the TRIzol reagent (T9424, Sigma) according to the manufacturer’s instructions. The concentration and quality of the mRNA were determined spectrophotometrically. Reverse-transcription reactions were performed using the PrimeScript RT reagent kit (RR047, Takara) on an Applied Biosystems 7500 Real-time PCR Detection System using a SYBR Premix Ex Taq II Kit (RR820, Takara). The following primers for GAPDH, GDNF, and BDNF were used: GAPDH: 5′-GGT TGT CTC CTG CGA CTT CA-3′ and 3′-CCT CAT TCT TTG GGA CCT GGT-5′; BDNF: 5′-GAG GTC TGA CGA CGA CAT CA-3′ and 3′-GTC AGT TCA CGG AA A CCT CG-5′; and GDNF:5′-CAA TGG ATT CAT ACC CTG-3′ and 3′-TCC AGA TAA TGT AGG TCG T-5′.All the samples were evaluated in triplicate, and averaged values were used for the 2^−ΔΔCT^ relative quantification.

### Western blot analysis

Western blot analysis was performed as reported previously^49^. The SNpc tissues were removed onto an ice-cold plate, lysed with a mixture containing RIPA and PMSF associated with phosphatase inhibitors, and then centrifuged at 13,500 rpm for 20 min at 4 °C. An equivalent amount of proteins was separated using SDS-PAGE gels and PVDF membranes. The membranes were blocked with 5% non-fat milk at room temperature for 1 h and then incubated overnight with primary antibodies against GDNF (1:1,000) and BDNF (1:1,000). Next, the membranes were incubated with a HRP-conjugated antibody against rabbit (dilution 1:1,000). The Immunoreactive bands were detected using enhanced chemiluminescence (P0018, Beyotime) and quantified using Image J software.

### Enzyme-linked immunosorbent assay measurement

The lipid peroxidation end-product MDA (Catalog #K739, BioVision, USA) and the antioxidative enzymes SOD (Catalog#K335, BioVison), CAT (Catalog #K773, BioVision), and GSH (Catalog #K261, BioVision) were detected using ELISA kits according to the manufacturer’s instructions.

### Ultrastructural alterations of the BBB

Mice were deeply anesthetized and then transcardially perfused with cold PBS. SNpc tissues were removed and divided into 1-mm^3^ segments. The samples were post fixed with 2.5% glutaraldehyde and 1% osmium for 2 h, followed by dehydration in a graded ethanol series and resin embedding. Ultrathin sections were sliced using a microtome, stained with 3% uranyl acetate-lead citrate and then observed with a TEM (PHILIPS CM-120).

### Evaluation of BBB permeability

BBB permeability was evaluated by the leakage of Evans Blue (E2129, Sigma) into the brain after tail vein injection as described previously^50^. Briefly, 2% Evans Blue in normal saline was injected 1 h before the animals were euthanized. The mice were deeply anesthetized and transcardially perfused with normal saline. SNpc tissues were dissected and incubated in formamide (100 mg/ml) at 60 °C for 24 h, followed by centrifugation at 1,000 rpm for 5 min. The supernatants were collected, and the absorption of the tissues was detected using a luminescence spectrometer at a wavelength of 620 nm based on the standard curve of Evans Blue.

### Statistical analysis

The data were analyzed using the SPSS 22.0 software employing a one-way analysis of variance (ANOVA) and using the post-hoc least significant difference (LSD) test. The results were expressed as the mean ± standard error for multiple group comparisons. Differences were considered statistically significant only when the *P-*value was less than 0.05 (*P* < 0.05).

## Electronic supplementary material

Dataset 1
