# Supplementary material for: Jia-Jian-Di-Huang-Yin-Zi decoction exerts neuroprotective effects on dopaminergic neurons and their microenvironment
Source: Sci Rep. 2018 Jun 29;8:9886. doi: 10.1038/s41598-018-27852-w (PMC6026152; doi:10.1038/s41598-018-27852-w)
Supplement: Supplementary file 1 — Dataset 1 [file 41598_2018_27852_MOESM1_ESM.doc]

Supplementary Figure.1

**Title:**

Jia-Jian-Di-Huang-Yin-Zi decoction exerts neuroprotective effects on dopaminergic neurons and their microenvironment

**The author list:**

Jingsi Zhang^a#^, Zhennian Zhang^b#^, Wen Zhang^a#^, Xiangting Li^a^, Ting Wu^a^, Tingting Li^c^, Min Cai^a^, Zhonghai Yu^a^, Jun Xiang^a^*, Dingfang Cai^a^*

1. Department of Integrative Medicine, Zhongshan Hospital, Fudan University, Shanghai 200032, China.

b. Department of Neurology, Nanjing Hospital of Traditional Chinese Medicine, Nanjing 210000, China.

c. Department of Neurology, Shuguang Hospital, Shanghai University of Traditional Chinese Medicine, Shanghai 201203, China.

#Jingsi Zhang, Zhennian Zhang and Wen Zhang have contributed equally to this work.

*Corresponding author:

E-mail: [dingfangcai@163.com(D](mailto:dingfangcai@163.com(Dingfang) Cai); [xiang.jun@mail.zs-hospital.sh.cn](mailto:xiang.jun@mail.zs-hospital.sh.cn) (J Xiang).

Tel: 86-021-64041990-3444

**Original figures:**

The grouping gels cropped from different gels for their molecular band sizes are too close to detect them in the same gel at the same time, and all the bands were exposured under the same condition.
**BDNF:**


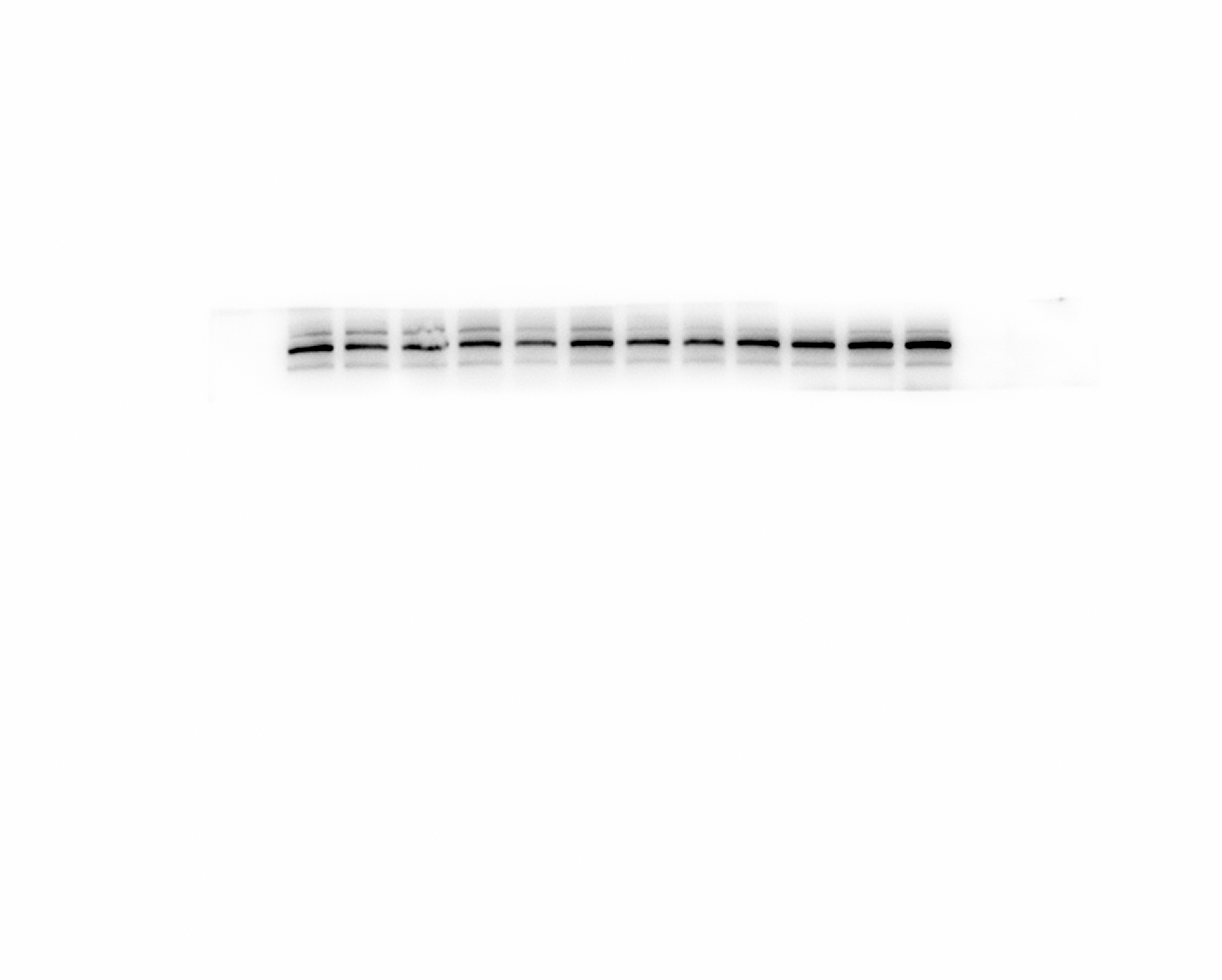


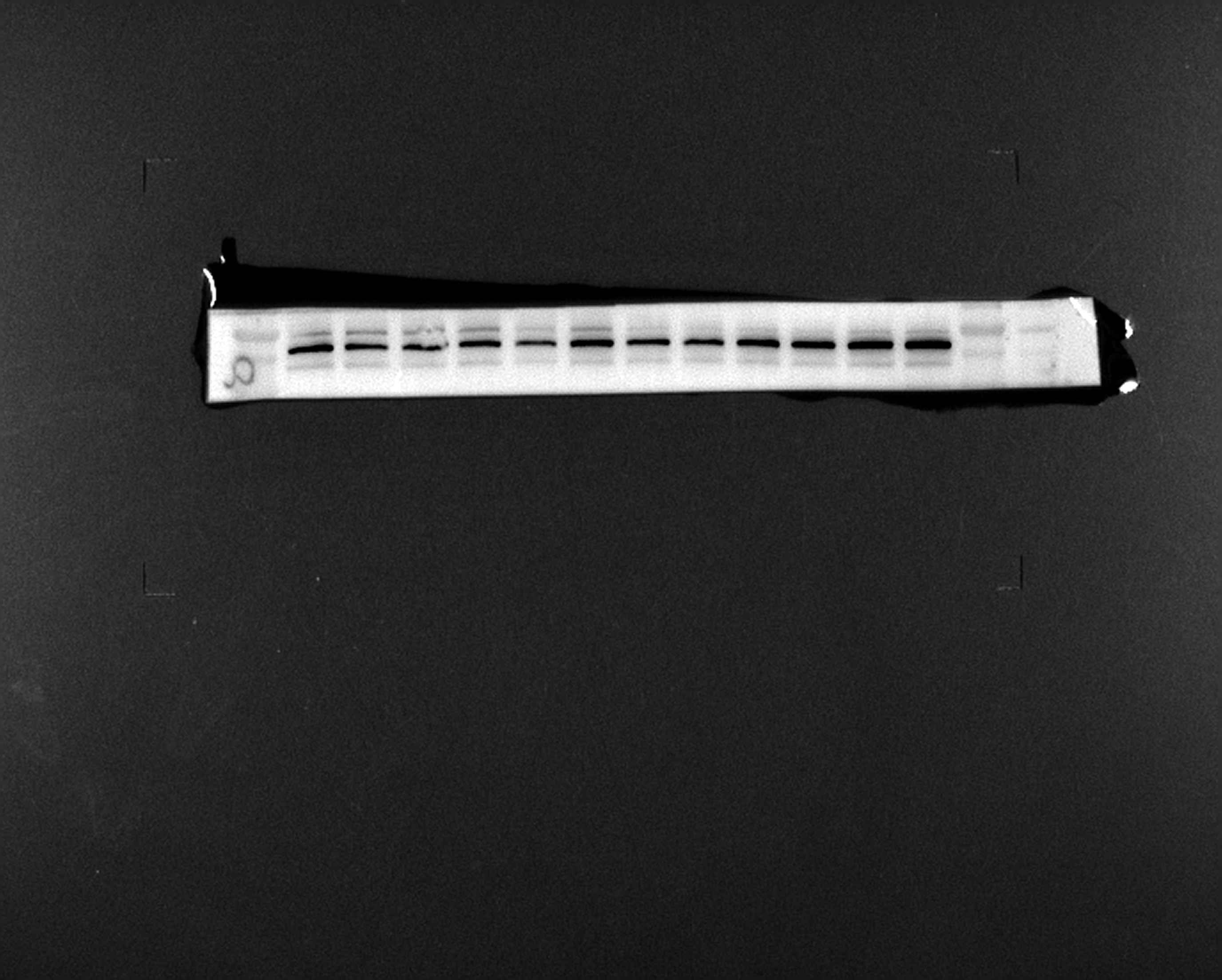


marker

Sample 1

NS M JL JM JH SE

NS M JL JM JH SE

Sample 2

marker

35KD

25KD

15KD

**GDNF:**


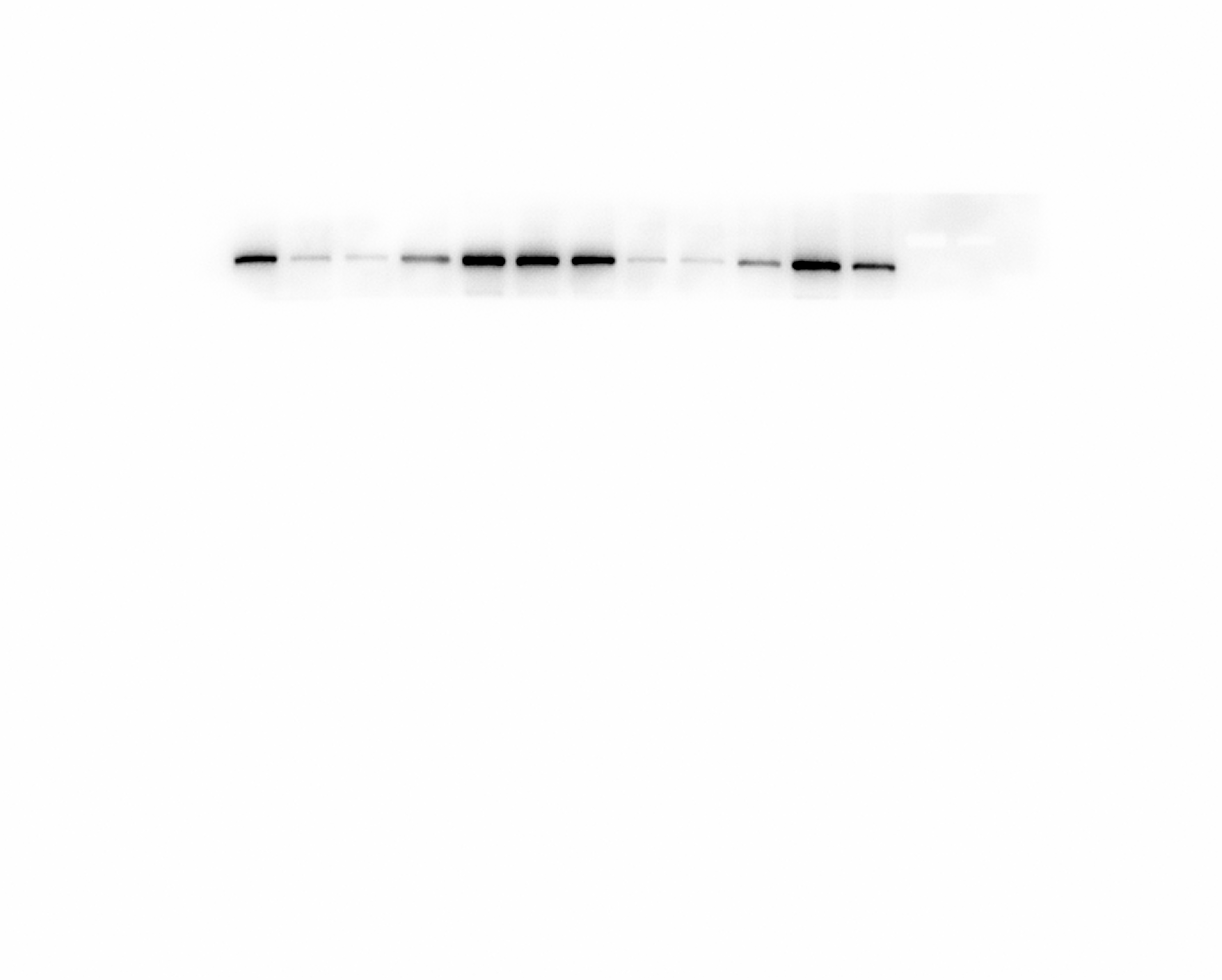


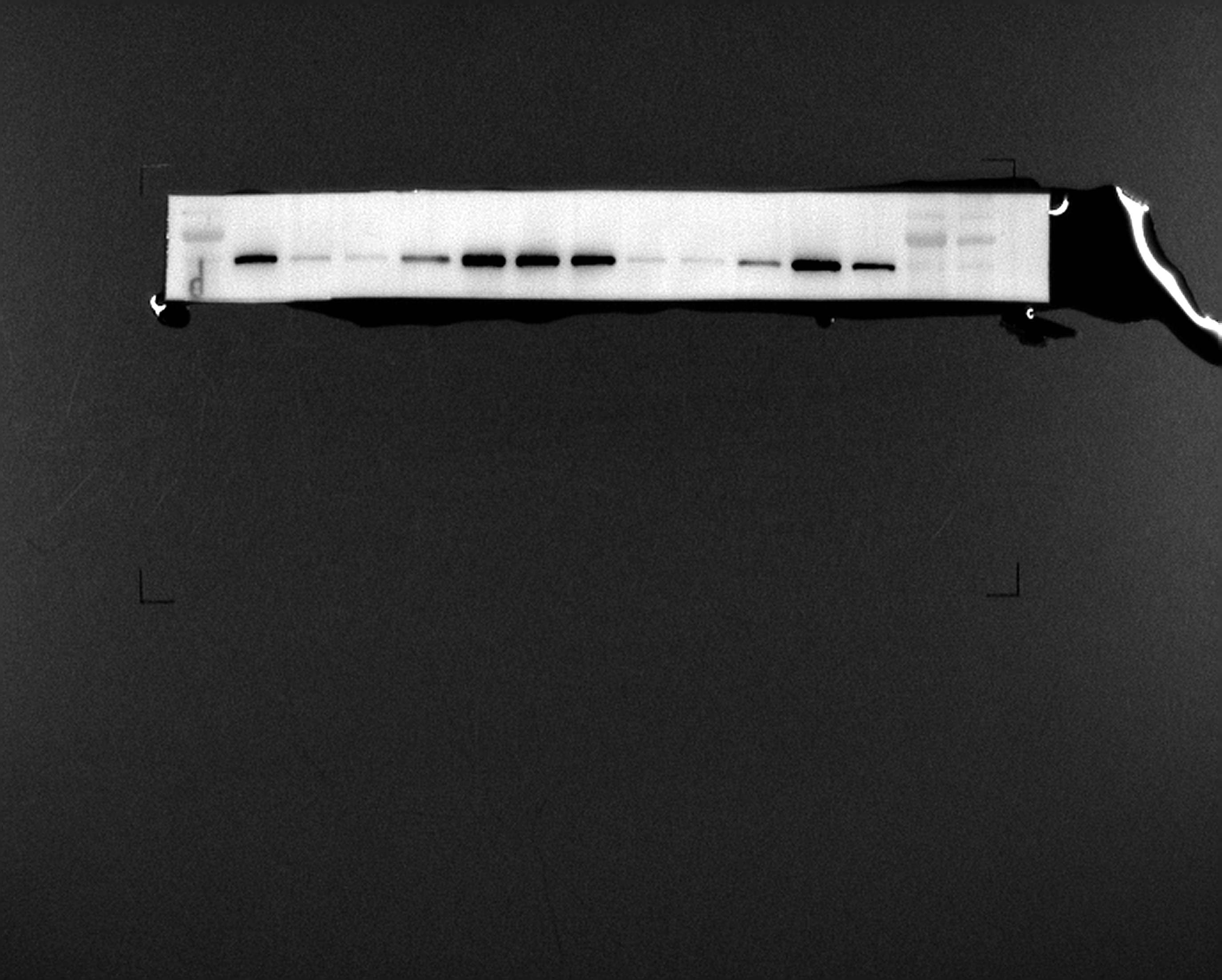


35KD

25KD

marker

Sample 1

NS M JL JM JH SE

NS M JL JM JH SE

Sample 2

marker

**GAPDH:**


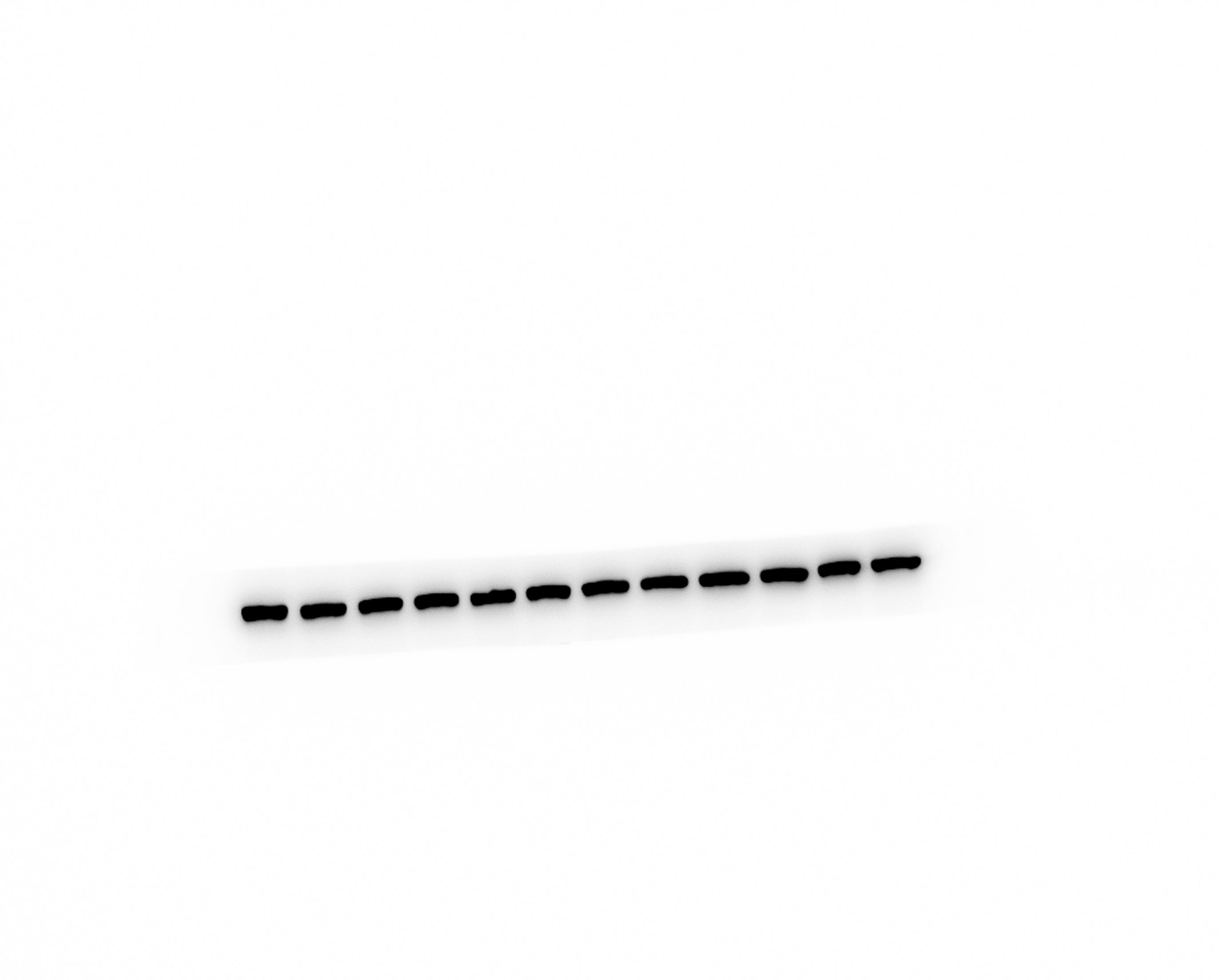


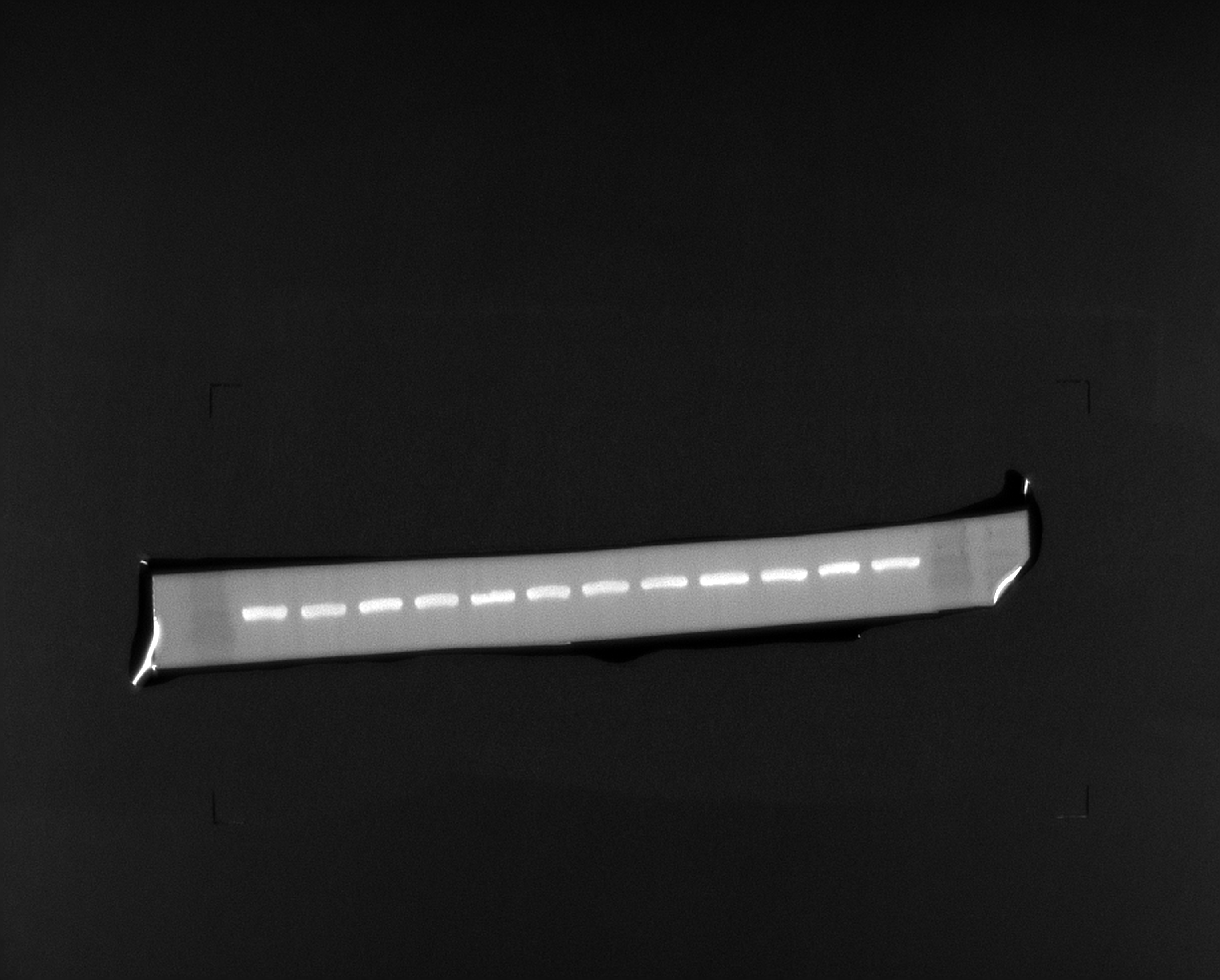


marker

35KD

Sample 1

NS M JL JM JH SE

NS M JL JM JH SE

Sample 2

marker
